# Insulin Sensitizers for Improving the Endocrine and Metabolic Profile in Overweight Women With PCOS

**DOI:** 10.1210/clinem/dgaa337

**Published:** 2020-06-03

**Authors:** Chuan Xing, Chunzhu Li, Bing He

**Affiliations:** The First Endocrinology Department of Shengjing Hospital of China Medical University, Shenyang

**Keywords:** polycystic ovary syndrome (PCOS), metformin, glucagon-like peptide-1 (GLP-1) receptor agonists, thiazolidinediones (TZDs), sex hormone, obesity

## Abstract

**Objective:**

To evaluate the efficacy of insulin sensitizers on menstrual frequency, sex hormone, and metabolic parameters in overweight women with polycystic ovary syndrome (PCOS).

**Methods:**

We searched multiple databases from inception to September 2019 for randomized controlled trials. Network meta-analysis was conducted using multivariate random effects method.

**Results:**

Fourteen trials reporting on 619 women were included. Compared with metformin, metformin + thiazolidinediones (TZDs) was more superior in menstrual recovery (weighted mean difference [WMD] 3.68; 95% credibility interval [CrI], 1.65 to 8.20), metformin +  glucagon-like peptide-1 (GLP-1) receptor agonists was more effective in decreasing androstenedione (WMD −2.53; 95% CrI, −3.96 to −1.09), both metformin + GLP-1 receptor agonists (WMD 9.22; 95% CrI, 5.46 to 12.98) and metformin + TZDs (WMD 4.30; 95% CrI, 0.78 to 7.82) were more effective in increasing sex hormone–binding globulin (SHBG), while TZDs were less effective in decreasing body mass index (BMI) (WMD 1.69; 95% CrI, 0.72 to 2.66). Compared with GLP-1 receptor agonists, metformin + GLP-1 receptor agonists was associated with higher SHBG (WMD 7.80; 95% CrI, 4.75 to 10.85), lower free testosterone (WMD −1.77; 95% CrI, −3.25 to −0.29), lower androstenedione (WMD −2.70; 95% CrI, −3.91 to −1.50) and lower fasting blood glucose (WMD −0.41; 95% CrI, −0.73 to −0.08).

**Conclusion:**

For overweight women with PCOS, both metformin combined with GLP-1 receptor agonists and metformin combined with TZDs appear superior to monotherapy in improving hyperandrogenemia. Metformin combined with TZDs could be particularly effective in promoting the recovery of menstruation. Metformin combined with GLP-1 receptor agonists has the additional advantage of improving fasting glucose when compared with GLP-1 receptor agonists alone. TZDs are inferior to metformin in decreasing BMI.

Polycystic ovary syndrome (PCOS) is characterized by oligoovulation or anovulation, elevated androgen production, and polycystic ovary changes observed by ultrasound. PCOS affects up to 13% of women of reproductive age (1). The pathogenesis of PCOS is far from completely clear and may relate to hyperandrogenemia, hyperinsulinemia, an imbalanced ratio of luteinizing hormone (LH) to follicle-stimulating hormone (FSH), metabolic aberrances, inflammation, advanced glycation end products, and endoplasmic reticulum stress (2-5). PCOS is often accompanied by obesity and increased rates of type 2 and gestational diabetes, cardiovascular disease, hepatic steatosis, and endometrial cancer, which, as long-term risks, can lead to serious threats to a woman’s life (6-9). Overweight women with PCOS are more prone to insulin resistance, which could lead to abnormal glucose and lipid metabolism (10). Moreover, hyperinsulinemia reduces the circulating level of sex hormone-binding globulin (SHBG) and increases free testosterone (FT), which inhibits follicular maturation with consequent menstrual irregularity and infertility (11).

Currently, symptomatic treatment dominates the clinical approach to PCOS and mainly includes the regulation of menstrual cycle, anti-hyperandrogenemia therapy, weight control, management of insulin resistance and metabolic disorders, and lifestyle modification (12-14). Drugs often used to reduce insulin resistance in obese patients with PCOS include metformin, glucagon-like peptide-1 (GLP-1) receptor agonists, and thiazolidinediones (TZDs) (7). However, the efficacy of different interventions on metabolism for PCOS remains to be verified. For this reason, we designed this meta-analysis to compare the effects of these drugs on the improvement of menstrual frequency, as well as sex hormone and metabolic parameters, of overweight women with PCOS. In addition, we searched for eligible interventions that can reduce hyperinsulinemia and induce weight loss in PCOS.

## Materials and Methods

### Literature search

This network meta-analysis (NMA) was registered with the PROSPERO international prospective register of systematic reviews (registration number CRD 156677). We searched medical literature for relevant studies using PubMed, EMBASE, the Cochrane Library, the WanFang Database, the WeiPu Database, and China National Knowledge Infrastructure (CNKI) from their dates of establishment to September 2019. A total of 5050 records were identified using electronic search strategies; in addition, 2 relevant records were obtained from the reference lists of the included studies. We used the search terms *polycystic ovary syndrome* with *metformin*, *glucagon-like peptide-1 receptor agonists*, and *thiazolidinediones* and limited the publication type to randomized controlled trials (RCTs). There were no language or location restrictions.

### Inclusion and exclusion criteria

To be included in this NMA, the studies needed to meet the following inclusion criteria: (1) women were diagnosed with PCOS, aged 18 to 49 years, with the lower inclusion limit of body mass index (BMI) ≥25 kg/m^2^; (2) the diagnosis of PCOS was based on the Rotterdam European Society for Human Reproduction and Embryology/American Society for Reproductive Medicine standard or the National Institute of Child Health and Human Development standard with no limits in terms of disease duration or ethnicity; (3) RCT study design; (4) the study included at least one of the outcomes of menstrual frequency, sex hormone parameters (including total testosterone [TT], FT, SHBG and androstenedione [AND]), glucose metabolism parameters (including fasting blood glucose [FG] and fasting insulin [FINS]) and obesity-related parameters (including BMI and waist circumference [WC]); (5) comparisons between the relevant interventions, including metformin, GLP-1 receptor agonists, TZDs, metformin + GLP-1 receptor agonists, and metformin + TZDs; and (6) the intervention period was at least 12 weeks.

Exclusion criteria included the following: (1) combinations with other related drugs, such as ovulation-inducing agents or other contraceptives; or (2) patients with other diseases such as congenital adrenal hyperplasia, Cushing syndrome, androgen-secreting neoplasm, hyperprolactinemia, diabetes, and kidney or liver disease.

### Outcomes, data extraction, and quality assessment

Outcomes included changes in menstrual frequency, sex hormone, glucose metabolism, and obesity-related parameters. Data were extracted according to study, author, year, region, size, interventions, follow-up, and efficacy (Table 1).

**Table 1.** Characteristics of Studies Included in the Network Meta-Analysis

Study	Year	Region	Size	Interventions	Follow-up	Efficacy*
Elkind-Hirsch (15)	2008	USA	14/14/14	MET vs GLP-1 vs MET + GLP-1	24 weeks	1, 2, 4, 8, 9
Jensterle (16)	2008	Slovenia	15/11	MET vs TZDs	24 weeks	1, 2, 3, 5, 6, 7, 8, 9
Jensterle (17)	2014	Slovenia	14/11/11	MET vs GLP-1 vs MET + GLP-1	12 weeks	1, 2, 3, 5, 8, 9
Jensterle (18)	2015	Slovenia	14/14	MET vs GLP-1	12 weeks	1, 2, 3, 4, 5, 8, 9
Jensterle (19)	2015	Slovenia	13/14	MET vs GLP-1	12 weeks	2, 3, 4, 5, 6, 7, 8, 9
Jensterle (20)	2016	Slovenia	21/22	GLP-1 vs MET + GLP-1	12 weeks	2, 3, 4, 5, 6, 7, 8, 9
Jensterle (21)	2017	Slovenia	14/14	GLP-1 vs MET + GLP-1	12 weeks	2, 3, 4, 5, 6, 7, 8, 9
Li (22)	2017	China	39/39	MET vs MET + TZDs	12 weeks	1, 2, 6, 7
Liang (23)	2019	China	22/21/23	MET vs TZDs vs MET + TZDs	12 weeks	1, 2, 6, 7, 8
Ortega-Gonzalez (24)	2005	Mexico	18/17	MET vs TZDs	24 weeks	3, 5, 6, 7, 8
Shahebrahimi (25)	2016	Iran	28/28	MET vs TZDs	12 weeks	1, 2, 6, 7, 8, 9
Steiner (26)	2007	Germany	16/17	MET vs TZDs	24 weeks	1, 6, 7, 8
Wang (27)	2014	China	41/40	MET vs MET + TZDs	24 weeks	1, 2, 4, 6, 7, 8
Yilmaz (28)	2005	Turkey	20/20	MET vs TZDs	12 weeks	1, 3, 5, 6, 7, 8, 9

Abbreviations: GLP-1, glucagon-like peptide-1 receptor agonist; MET, metformin; TZD, thiazolidinedione.

*Efficacy: 1, Menstrual frequency; 2, Total Testosterone (TT); 3, Free Testosterone (FT); 4, Sex hormone binding globulin (SHBG); 5, Androstenedione (AND); 6, Fasting glucose (FG); 7, Fasting insulin (FINS); 8, Body mass index (BMI); 9, Waist circumference (WC).

The included trials were assessed for quality by 2 authors (X.C. and L.C.Z.) using Cochrane Risk of Bias Tool 2.0 for RCTs (29). We assessed the methodological quality of the included RCTs according to standard criteria of The Cochrane Collaboration. Seven domains related to risk of bias were assessed for each study, including: (1) random sequence generation; (2) allocation concealment; (3) blinding of participants and personnel; (4) blinding of outcome assessment; (5) incomplete outcome data; (6) selective reporting; and (7) other bias. Every question was answered “yes,” “no,” or “unclear,” and 2 reviewers assessed each trial. In case of a disagreement, judgment was made through open discussion. According to criteria of The Cochrane Collaboration, we divided the studies into 3 categories: (1) low risk of bias: low risk of bias for all key domains; (2) unclear risk of bias: unclear risk of bias for one or more key domains; and (3) high risk of bias: high risk of bias for one or more key domains. Discrepancies in risk assessments were discussed with the third author (H.B.).

### Statistical analysis

#### Data synthesis and analysis.

Both traditional meta-analysis (TMA) and NMA were performed to simultaneously compare the efficacies of different treatment options for managing changes in menstrual frequency, sex hormone parameters (including TT, FT, SHBG, and AND), metabolic parameters (including FG and FINS), and obesity-related parameters (including BMI and WC) (30). For dichotomous outcomes, we calculated pooled odds ratios (ORs) with 95% confidence intervals (CIs). For continuous outcomes, we calculated weighted mean differences (WMDs) with 95% CIs. All tests were two-tailed, and a *P* value < 0.05 was deemed statistically significant.

#### Traditional meta-analysis.

We performed TMA using RevMan version 5.3 (Nordic Cochrane Center) to directly compare the efficacies of metformin, GLP-1 receptor agonists, TZDs, metformin + GLP-1 receptor agonists, and metformin + TZDs. The WMD and 95% CI between 2 groups were synthesized using the mean difference (MD) and standard deviation (SD), and the sample size or the data were converted from the MD and sample SD at the endpoint between 2 groups (31). Interstudy heterogeneity between the trials was assessed using the chi-squared test for analysis, combining *I*^*2*^ and *P* value to determine the level of heterogeneity where *I*^*2*^* > *50% or *P* < 0.10 suggested a high level of heterogeneity. For all the parameters included in this study, the random effect models were used. Although we planned to assess publication bias by using the Egger regression asymmetry test, we were not able to conduct formal testing because of the small number of studies available from head-to-head comparisons. Funnel plots created by Stata software (version 15.0, Stata Corp, College Station, TX) were used to examine publication bias.

#### Network meta-analysis.

The NMA was performed to combine results from direct and indirect comparisons of treatment effect in a single analysis with Stata software (version 15.0, Stata Corp, College Station, TX). The analysis model was based on the definition of likelihood and prior probability, and we confirmed the convergence and calculated the pooled estimates of MDs and 95% credibility intervals (CrIs) to compare the 5 different interventions to each other. Additionally, the surface under the cumulative ranking (SUCRA) curve was calculated to rank the different interventions. Compared with other interventions, 1 intervention showed a higher SUCRA value, so it might have a greater effect on the endpoint (32). The consistency assumption of direct evidence and indirect evidence was assessed by the node-splitting method. When the results showed that the direct evidence of the outcomes was consistent with indirect evidence (*P* values > 0.05), the consistency model was adopted.

## Results

### Literature identification

**Figure 1.**
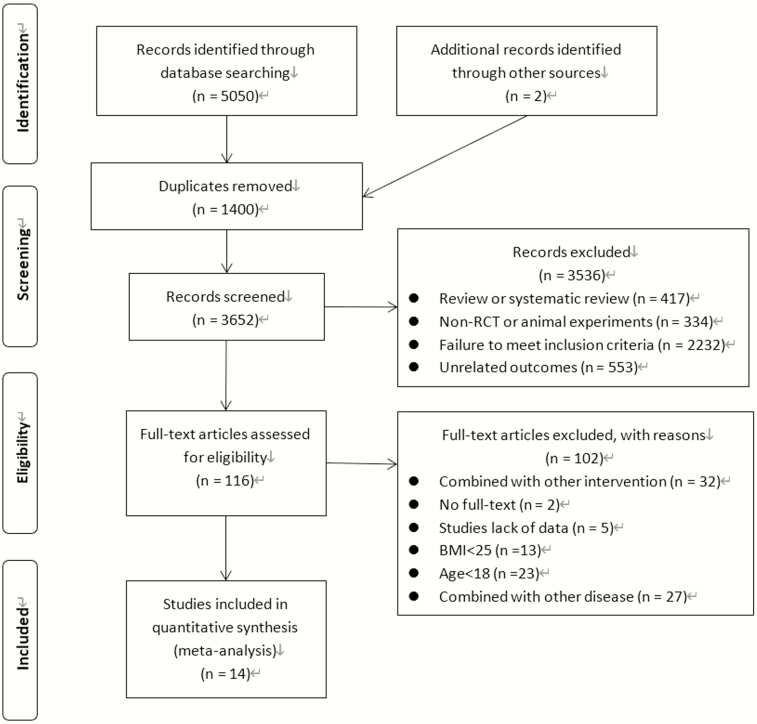
Flow diagram of studies identified in the systematic review. Abbreviations: BMI, body mass index (kg/m^2^); RCT, randomized controlled trial.

The initial search strategy retrieved a total of 5052 studies. After reading the titles, abstracts, and full texts, we selected 14 RCTs, which included a total of 619 women, for inclusion in the analysis. The remaining studies were excluded: 1400 were duplicates, 417 were reviews, 334 were non-RCTs or animal experiments, 2232 failed to meet the inclusion criteria, 553 included unrelated outcomes, 32 combined treatments with other interventions, 2 had no full text available, 5 lacked relevant data, 13 included participants with BMI <25 kg/m^2^, 23 included participants with age <18 years, and 27 combined with other diseases. The literature screening process and results are shown in Fig. 1.

### Characteristics of included studies

**Figure 2.**
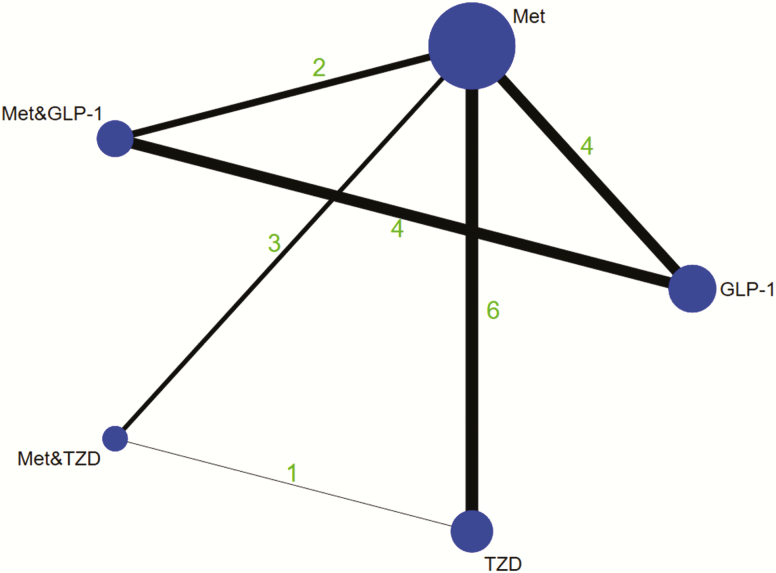
Network of eligible comparisons for efficacy. The size of the circles is proportional to sample size, and the width of the lines is proportional to the number of trials. Abbreviations: GLP-1, glucagon like peptide-1 receptor agonist; MET, metformin; TZD, thiazolidinedione.

Table 1 presents the characteristics of included studies. Trials were performed in European (16-21, 26), American (15, 24), and Asian (22, 23, 25, 27, 28) countries and were published from 2005 to 2019. Participants in these trials were recruited from outpatient clinics, hospitals, or medical centers and were diagnosed with PCOS according to either the National Institutes of Health or Rotterdam criteria. Participants aged 18 to 49 years were included in all 14 studies. In all, 5 different interventions were included: metformin, GLP-1 receptor agonists, TZDs, metformin + GLP-1 receptor agonists, and metformin + TZDs (shown in Fig. 2). Twelve studies evaluated the use of metformin alone, participants received a dosage of metformin ranging from 1.5 g/d to 3 g/d for 12 to 24 weeks. Six studies evaluated GLP-1 receptor agonists alone: 4 studies used liraglutide 1.2 mg/d for 12 weeks, 1 study used liraglutide 3.0 mg/d for 12 weeks, and 1 study used exenatide 20 µg/d for 24 weeks. Four studies evaluated GLP-1 receptor agonists in combination with metformin: 3 used liraglutide 1.2 mg/d in combination with metformin 2.0 g/d for 12 weeks and 1 used exenatide 20 µg/d in combination with metformin 2.0 g/d for 24 weeks. Six studies evaluated TZDs alone: 2 used rosiglitazone 4 mg/d for 24 weeks, 1 used rosiglitazone 4 mg/d for 12 weeks, 1 used pioglitazone 30 mg/d for 24 weeks, 1 used pioglitazone 30 mg/d for 12 weeks, and 1 used pioglitazone 90 mg/d for 12 weeks. Three studies evaluated TZDs in combination with metformin: 1 used pioglitazone 90 mg/d in combination with metformin 1.5 g/d for 12 weeks, 1 used pioglitazone 30 mg/d in combination with metformin 1.0 g/d for 12 weeks, and 1 used pioglitazone 15 mg/d in combination with metformin 3.0 g/d for 24 weeks. For studies included in our meta-analysis, the dropout rate was 7.64% (21/275) for metformin, 11.76% (12/102) for GLP-1 receptor agonists, 8.80% (11/125) for TZDs, 12.86% (9/70) for metformin + GLP-1 receptor agonists, and 2.86% (3/105) for metformin + TZDs.

### Quality assessment of the included studies

**Figure 3.**
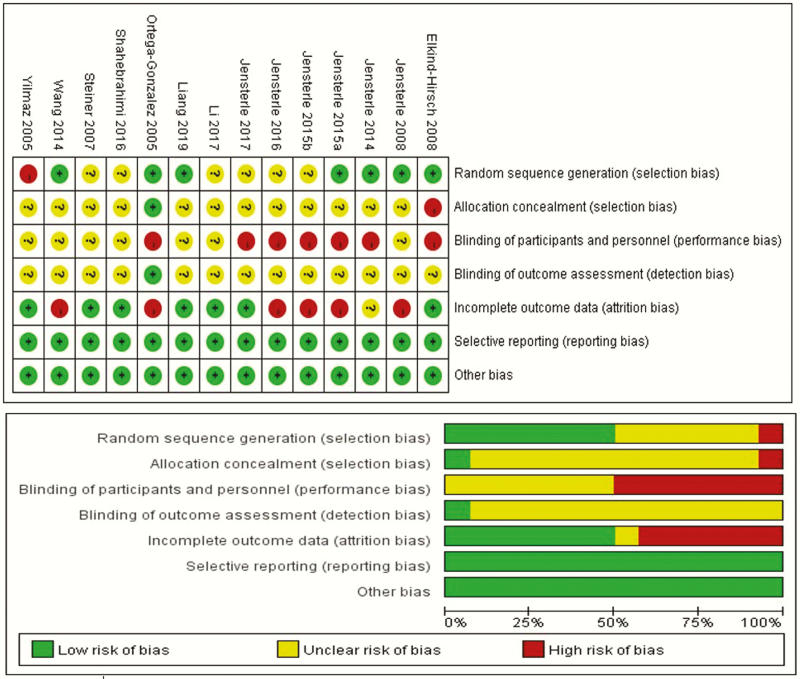
Risk of bias assessment in the RCTs.

Quality assessment was evaluated in Fig. 3, showing that the lack of blinding was the main cause of potential bias. In trials containing GLP-1 receptor agonists, blinding was not possible because of the different routes of administration of GLP-1 receptor agonists and metformin. In addition, incomplete outcome data was the second cause of potential bias because the number and cause of missing outcome indicators were not consistent between groups. All funnel plots showed minor asymmetry or no publication bias (shown in Figs. S1-S10) (55).

### Main outcomes

#### Improvement of menstrual frequency.

Data on improving menstrual frequency were reported, with 5 studies showing changes of menstrual cycle frequency (cycles per month) and 5 studies showing the number of menstrual recovery (shown in Table 2 and Figs. S11-S12 as menstrual frequency(a) and menstrual frequency(b), respectively) (55). TMA for menstrual frequency(a) revealed that no significant difference among GLP-1 receptor agonists, TZDs, and metformin, while metformin + GLP-1 receptor agonists (WMD 0.02; 95% CI, 0.02-0.02; *P* = 0.81, *I*^*2*^ = 0%) was more effective than GLP-1 receptor agonists alone. TMA for Menstrual frequency(b) revealed that metformin + TZDs (WMD 4.40; 95% CI, 1.95-9.96; *P* = 0.80, *I*^*2*^* = *0%) was more effective than metformin alone. Similarly, NMA for Menstrual frequency(b) also revealed that metformin + TZDs (WMD 3.68; 95% CrI, 1.65-8.20) was more effective than metformin.

**Table 2.** Results of NMA and TMA for Improving Menstrual Frequency

			TMA		NMA
Study Group	Studies	Participants	Heterogeneity	Effect Estimate (95% CI)	Effect Estimate (95% CrI)
Menstrual frequency(a)*	5				
GLP-1 vs MET	3	81	(*P* = 0.0005); *I*^*2*^ = 87%	0.04 (−0.16, 0.24)	0.04 (−0.14, 0.22)
TZD vs MET	2	59	(*P* = 0.77); *I*^*2*^* *= 0%	−0.01 (−0.11, 0.09)	−0.01 (−0.24, 0.21)
MET + GLP-1 vs MET	2	53	(*P* = 0.002); *I*^*2*^ = 89%	0.15 (−0.11, 0.41)	0.10 (−0.11, 0.30)
MET + GLP-1 vs GLP-1	2	50	(*P* = 0.81); *I*^*2*^ = 0%	***0.02 (0.02, 0.02)***	0.06 (−0.15, 0.26)
Menstrual frequency(b)**	5				
TZD vs MET	3	139	(*P* = 0.22); *I*^*2*^ = 35%	1.82 (0.76, 4.36)	1.80 (0.88, 3.67)
MET + TZD vs MET	3	204	(*P* = 0.80); *I*^*2*^ = 0%	***4.40 (1.95, 9.96)***	***3.68 (1.65, 8.20)***
MET + TZD vs TZD	1	44	Not applicable	1.13 (0.30, 4.27)	2.05 (0.77, 5.42)

Abbreviations: GLP-1, glucagon-like peptide-1 receptor agonist; MET, metformin; TZD, thiazolidinedione.

*Menstrual frequency(a) is menstrual cycle frequency (cycles per month).

** Menstrual frequency(b) is menstrual recovery.

#### Improvement of hyperandrogenemia.

For improving hyperandrogenemia, outcomes included TT, FT, SHBG, and AND (shown in Table 3 and Figs. S13-S16) (55).

**Table 3.** Results of NMA and TMA for Improving Hyperandrogenemia

			TMA		NMA
Study Group	Studies	Participants	Heterogeneity	Effect Estimate (95% CI)	Effect Estimate (95% CrI)
**TT**	11				
GLP-1 vs MET	4	108	*(P < 0.0001); I* ^*2*^ * = 88%*	0.26 (−0.28, 0.79)	0.18 (−0.27, 0.63)
TZD vs MET	3	125	*(P = 0.06); I* ^*2*^ * = 64%*	0.01 (−0.02, 0.05)	0.01 (−0.47, 0.50)
MET + GLP-1 vs MET	2	53	*(P = 0.48); I* ^*2*^ * = 0%*	***−0.51 (−0.63, −0.39)***	−0.19 (−0.72, 0.35)
MET + TZD vs MET	3	204	*(P < 0.00001); I* ^*2*^ * = 96%*	***−0.37 (−0.74, 0.00)***	−0.36 (−0.85, 0.12)
MET + GLP-1 vs GLP-1	4	121	*(P = 0.22); I* ^*2*^ * = 32%*	***−0.27 (−0.48, −0.06)***	−0.36 (−0.82, 0.09)
MET + TZD vs TZD	1	44	*Not applicable*	0.00 (−0.03, 0.03)	−0.38 (−0.98, 0.23)
**FT**	8				
GLP-1 vs MET	3	80	*(P = 0.02); I* ^*2*^ * = 74%*	0.61 (−1.12, 2.33)	0.55 (−0.73, 1.84)
TZD vs MET	3	101	*(P = 0.16); I* ^*2*^ * = 46%*	−0.29 (−1.61, 1.02)	−0.29 (−1.65, 1.07)
MET + GLP-1 vs MET	1	25	*Not applicable*	−1.30 (−3.01, 0.41)	−1.22 (−2.95, 0.52)
MET + GLP-1 vs GLP-1	3	93	*(P = 0.64); I* ^*2*^ * = 0%*	***−1.84 (−2.95, −0.73)***	***−1.77 (−3.25,−0.29)***
**SHBG**	6				
GLP-1 vs MET	3	83	*(P = 0.60); I* ^*2*^ * = 0%*	1.08 (−1.56, 3.72)	1.42 (−1.76, 4.60)
MET + GLP-1 vs MET	1	28	*Not applicable*	***10.90 (7.66, 14.14)***	***9.22 (5.46, 12.98)***
MET + TZD vs MET	1	81	*Not applicable*	***4.30 (1.70, 6.90)***	***4.30 (0.78, 7.82)***
MET + GLP-1 vs GLP-1	3	99	*(P = 0.38); I* ^*2*^ * = 0%*	***7.44 (4.82, 10.07)***	***7.80 (4.75, 10.85)***
**AND**	8				
GLP-1 vs MET	3	80	*(P = 0.31); I* ^*2*^ * = 15%*	0.37 (−1.03, 1.77)	0.18 (−1.00, 1.36)
TZD vs MET	3	101	*(P = 0.09); I* ^*2*^ * = 58%*	0.03 (−0.16, 0.21)	0.03 (−0.16, 0.21)
MET + GLP-1 vs MET	1	25	*Not applicable*	***−3.00 (−4.99, −1.01)***	***−2.53 (−3.96,−1.09)***
MET + GLP-1 vs GLP-1	3	93	*(P = 0.52); I* ^*2*^ * = 0%*	***−2.74 (−3.99, −1.48)***	***−2.70 (−3.91,−1.50)***

Abbreviations: AND, androstenedione; FT, free testosterone; GLP-1, glucagon-like peptide-1 receptor agonist; MET, metformin; SHBG, sex hormone–binding globulin; TT, total testosterone; TZD, thiazolidinedione.

Data on decreasing TT were reported in 11 studies. TMA revealed that no significant difference among GLP-1 receptor agonists, TZDs, and metformin while both metformin + GLP-1 receptor agonists (WMD −0.51; 95% CI, −0.63 to −0.39; *P* = 0.48, *I*^*2*^ = 0%) and metformin + TZDs (WMD −0.37; 95% CI, −0.74 to 0.00; *P* < 0.00001, *I*^*2*^ = 96%) were more effective than metformin alone. TMA also showed that metformin + GLP-1 receptor agonists (WMD −0.27; 95% CI, −0.48 to −0.06; *P* = 0.22, *I*^*2*^ = 32%) were more effective than GLP-1 receptor agonists alone. However, NMA showed no significant difference between groups.

As for decreasing FT, TMA revealed that no significant difference on GLP-1 receptor agonists, TZDs, and metformin + GLP-1 receptor agonists when compared with metformin alone while metformin + GLP-1 receptor agonists (WMD −1.84; 95% CI, −2.95 to −0.73; *P* = 0.48, *I*^*2*^* = *0%) was more effective than GLP-1 receptor agonists alone. The NMA also revealed that metformin + GLP-1 receptor agonists (WMD −1.77; 95% CrI, −3.25 to −0.29) was more effective than GLP-1 receptor agonists.

For increasing SHBG, TMA revealed no significant difference between GLP-1 receptor agonists and metformin, while both metformin + GLP-1 receptor agonists (WMD 10.90; 95% CI, 7.66-14.14, *P*&*I*^*2*^ not applicable [NA]) and metformin + TZDs (WMD 4.30; 95% CI, 1.70-6.90, *P*&*I*^*2*^ NA) were more effective than metformin alone. TMA also showed that metformin + GLP-1 receptor agonists (WMD 7.44; 95% CI, 4.82-10.07; *P* = 0.38, *I*^*2*^ = 0%) were more effective than GLP-1 receptor agonists alone. Similarly, the NMA revealed that both metformin + GLP-1 receptor agonists (WMD 9.22; 95% CrI, 5.46-12.98) and metformin + TZDs (WMD 4.30; 95% CrI, 0.78-7.82) were more effective than metformin alone and metformin + GLP-1 receptor agonists (WMD 7.80; 95% CrI, 4.75-10.85) was more effective than GLP-1 receptor agonists alone.

For data on decreasing AND, TMA revealed that there was no significant difference among GLP-1 receptor agonists, TZDs, and metformin while metformin + GLP-1 receptor agonists was more effective than metformin (WMD −3.00; 95% CI, −4.99 to −1.01, *P*&*I*^*2*^ NA) or GLP-1 receptor agonists alone (WMD −2.74; 95% CI, −3.99 to −1.48; *P* = 0.52, *I*^*2*^ = 0%). NMA also revealed that metformin + GLP-1 receptor agonists was more effective than metformin (WMD −2.53; 95% CrI, −3.96 to −1.09) or GLP-1 receptor agonists alone (WMD −2.70; 95% CrI, −3.91 to −1.50).

#### Improvement of glucose metabolism.

For improving glucose metabolism, outcomes included FG and FINS (shown in Table 4 and Figs. S17-S18) (55).

**Table 4.** Results of NMA and TMA for Improving Glucose Metabolism

			TMA		NMA
Study Group	Studies	Participants	Heterogeneity	Effect Estimate (95% CI)	Effect Estimate (95% CrI)
**FG**	11				
GLP-1 vs MET	1	27	Not applicable	0.00 (−0.66, 0.66)	0.00 (−0.68, 0.68)
TZD vs MET	6	235	(*P* = 0.31); I^2^ = 16%	***0.07 (0.02, 0.11)***	0.02 (−0.21, 0.25)
MET + GLP-1 vs MET	0	0	/	/	0.08 (−0.02, 0.18)
MET + TZD vs MET	3	204	(*P* < 0.0001); I^2^ = 89%	−0.05 (−0.16, 0.06)	−0.41 (−1.16, 0.35)
MET + GLP-1 vs GLP-1	2	71	(*P* = 0.36); I^2^ = 0%	***−0.40 (−0.71, −0.10)***	***−0.41 (−0.73,−0.08)***
MET + TZD vs TZD	1	44	Not applicable	0.10 (−0.03, 0.23)	0.12 (−0.13, 0.37)
**FINS**	11				
GLP-1 vs MET	1	27	Not applicable	1.30 (−2.55, 5.15)	1.30 (−2.55, 5.15)
TZD vs MET	6	235	(*P* = 0.54); I^2^ = 0%	−0.01 (−0.03, 0.01)	−0.01 (−0.03, 0.01)
MET + GLP-1 vs MET	0	0	/	/	3.38 (−2.07, 8.83)
MET + TZD vs MET	3	204	(*P* = 0.02); I^2^ = 74%	−0.16 (−2.55, 2.23)	0.03 (−0.72, 0.79)
MET + GLP-1 vs GLP-1	2	71	(*P* = 0.11); I^2^ = 60%	0.30 (−7.80, 8.39)	2.08 (−1.78, 5.93)
MET + TZD vs TZD	1	44	Not applicable	−0.60 (−3.31, 2.11)	0.04 (−0.71, 0.80)

Abbreviations: FG, fasting blood glucose; FINS, fasting insulin; GLP-1, glucagon-like peptide-1 receptor agonist; MET, metformin; TZD, thiazolidinedione.

Data on decreasing FG were reported in 11 studies. TMA revealed that there was no significant difference between GLP-1 receptor agonists and metformin, while TZDs (WMD 0.07; 95% CI, 0.02-0.11; *P* = 0.31, *I*^*2*^ = 16%) were less effective than metformin. Both TMA (WMD −0.40; 95% CI, −0.71 to −0.10; *P* = 0.36, *I*^*2*^* = *0%) and NMA (WMD −0.41; 95% CrI, −0.73 to −0.08) showed that metformin + GLP-1 receptor agonists was more effective than GLP-1 receptor agonists alone.

For data on decreasing FINS, both TMA and NMA revealed no significant difference between groups.

#### Improvement of obesity.

For improving obesity, outcomes included BMI and WC (shown in Table 5 and Figs. S19-S20) (55).

**Table 5.** Results of NMA and TMA for Improving Obesity

			TMA		NMA
Study Group	Studies	Participants	Heterogeneity	Effect Estimate (95% CI)	Effect Estimate (95% CrI)
**BMI**	13				
GLP-1 vs MET	4	108	(*P* = 0.87); I^2^ = 0%	−0.38 (−1.41, 0.64)	−0.61 (−1.86, 0.64)
TZD vs MET	6	235	(*P* = 0.006); I^2^ = 69%	***1.52 (0.28, 2.75)***	***1.69 (0.72, 2.66)***
MET + GLP-1 vs MET	2	53	(*P* = 0.44); I^2^ = 0%	−1.02 (−2.27, 0.24)	−0.85 (−2.22, 0.52)
MET + TZD vs MET	2	126	(*P* = 0.21); I^2^ = 38%	0.25 (−0.94, 1.43)	0.40 (−0.96, 1.75)
MET + GLP-1 vs GLP-1	4	121	(*P* = 0.01); I^2^ = 73%	−0.26 (−1.33, 0.81)	−0.24 (−1.22, 0.74)
MET + TZD vs TZD	1	44	Not applicable	−1.10 (−2.63, 0.43)	−1.30 (−2.81, 0.22)
**WC**	9				
GLP-1 vs MET	4	108	(*P* = 0.90); I^2^ = 0%	−1.57 (−3.30, 0.17)	1.66 (−3.49, 6.82)
TZD vs MET	3	122	(*P* = 0.65); I^2^ = 0%	1.74 (−0.94, 4.42)	−2.99 (−7.79, 1.82)
MET + GLP-1 vs MET	2	53	(*P* = 0.50); I^2^ = 0%	***−6.31 (−8.27, −4.35)***	1.74 (−0.94, 4.42)
MET + GLP-1 vs GLP-1	4	121	(*P* = 0.009); I^2^ = 74%	−2.67 (−7.25, 1.91)	−3.36 (−8.55, 1.82)

Abbreviations: BMI, body mass index; GLP-1, glucagon-like peptide-1 receptor agonist; MET, metformin; TZD, thiazolidinedione; WC, waist circumference.

Data on decreasing BMI were reported in 13 studies. NMA revealed no significant difference among GLP-1 receptor agonists, metformin + GLP-1 receptor agonists, and metformin + TZDs, when compared with metformin alone. Both TMA (WMD 1.52; 95% CI, 0.28 to 2.75; *P* = 0.006, *I*^*2*^* = *69%) and NMA (WMD 1.69; 95% CrI, 0.72-2.66) revealed that TZDs were less effective than metformin.

For data on decreasing WC, both TMA and NMA revealed no significant difference among GLP-1 receptor agonists, TZDs, and metformin, while TMA showed that metformin + GLP-1 receptor agonists (WMD −6.31; 95% CI, −8.27 to −4.35; *P* = 0.50, *I*^*2*^* = *0%) was more effective than metformin alone.

#### SUCRA.

The SUCRA curve of outcomes illustrated that metformin + GLP-1 receptor agonists was the best intervention for improving menstrual frequency(a) (SUCRA 75.5), FT (SUCRA 90.3), SHBG (SUCRA 99.1), AND (SUCRA 100), FG (SUCRA 85.4), BMI (SUCRA 86.4), and WC (SUCRA 100); metformin + TZDs was the best intervention in improving menstrual frequency(b) (SUCRA 96.1) and TT (SUCRA 85.9), while TZDs were the best intervention for improving FINS (SUCRA 76.8) (shown in Table 6).

**Table 6.** SUCRA Values and Ranks of Efficacy Outcomes

	Menstrual frequency(a)		Menstrual frequency(b)		TT		FT		SHBG	
Interventions	SUCRA	Rank	SUCRA	Rank	SUCRA	Rank	SUCRA	Rank	SUCRA	Rank
Met	35.4	4	2.8	3	40.3	3	40.9	3	6.6	4
GLP-1	53.6	2	/	/	16.6	5	13.5	4	30.9	3
TZD	35.5	3	51.1	2	38.9	4	55.3	2	/	/
Met + GLP-1	***75.5***	1	/	/	68.2	2	***90.3***	1	***99.1***	1
Met + TZD	/	/	***96.1***	1	***85.9***	1	/	/	63.4	2
Inventions	AND		FG		FINS		BMI		WC	
	SUCRA	Rank	SUCRA	Rank	SUCRA	Rank	SUCRA	Rank	SUCRA	Rank
Met	41.3	2	40.7	3	56.8	3	50	3	32.7	3
GLP-1	26.3	4	34.3	4	40.7	4	***75.3***	2	63.1	2
TZD	32.5	3	14.4	5	***76.8***	1	1.4	5	4.2	4
Met + GLP-1	***100***	1	***85.4***	1	12.3	5	***86.4***	1	***100***	1
Met + TZD	/	/	***75.2***	2	63.3	2	36.9	4	/	/

Bold and inclined type indicates that the SUCRA is relatively higher when compared with other interventions.

Abbreviations: AND, androstenedione; BMI, body mass index; FG, fasting glucose; FINS, fasting insulin; FT, free testosterone; GLP-1, glucagon-like peptide-1 receptor agonist; Met, metformin; PCOS, polycystic ovary syndrome; SHBG, sex hormone-binding globulin; SUCRA, surface under the cumulative ranking; TT, total testosterone; TZD, thiazolidinedione; WC, waist circumference.

## Discussion

This is the first NMA comparing efficacy and safety of both monotherapy and combinations of different insulin sensitizers in overweight PCOS. Many studies suggest that, in obese PCOS patients, metabolic abnormalities related to insulin resistance and obesity are more important than androgen excess in the mechanism of anovulation in PCOS (33). Metformin, which has insulin-lowering effects by ameliorating insulin sensitivity in liver and peripheral tissues, is the most widely used drug for regulating metabolic and endocrine disorders in PCOS, and it can, in turn, improve ovarian steroidogenesis and decrease circulating androgen level. These changes offer benefits that targets both cardiometabolic disorders and reproductive abnormalities (34). TZDs such as pioglitazone and rosiglitazone have also been shown to be effective in improving insulin resistance and hyperandrogenemia, as well as ovulation rate and menstrual cyclicity in PCOS, and GLP-1 receptor agonists such as exenatide and liraglutide are novel therapeutic options for the treatment of obesity, particularly in women with PCOS who display impaired first- and second-phase insulin secretion (34). Our results showed that metformin combined with GLP-1 receptor agonists was superior to monotherapy in improving SHBG and AND; in addition, it had better effects on reducing FT and FG when compared with GLP-1 receptor agonists alone. Metformin combined with TZDs was associated with better effects on increasing SHBG and promoting the recovery of menstruation than metformin. We did not observe a difference between metformin, GLP-1 receptor agonists, and TZD monotherapy in terms of menstrual frequency, hyperandrogenemia, FINS, and WC improvement; whereas, TZDs were inferior to metformin in decreasing BMI.

Menstrual disorders and symptoms of sterility occur in approximately 98% of women with PCOS (35). Metformin may improve menstrual cyclicity and ovulation; moreover, it may also amplify the effects of ovulation-inducing drugs or androgen-lowering medications (36). Other studies have shown that pioglitazone and GLP-1 receptor agonists could also restore menstrual cycles and induce ovulation (37, 38). We confirmed that all these interventions were effective; in addition, both TMA and NMA demonstrated that metformin combined with TZDs was more effective than metformin alone and the SUCRA curve illustrated that metformin combined with TZDs was the best intervention for promoting menstrual recovery.

For the improvement of hyperandrogenemia, metformin can reduce androgen secretion from thecal cells and adrenal glands of women with PCOS while stimulating SHBG production, modulating LH discharge, and attenuating the ovarian androgen response to gonadotropin stimulation by reducing circulating insulin and androgen levels, thus reducing FT concentration (39, 40). Both animal experiments and clinical research have shown that GLP-1 receptor agonists can reduce serum testosterone concentration in PCOS (41, 42), and TZDs were found to repress androgen biosynthesis in thecal cells (43). Both TMA and NMA showed no significant difference among metformin, GLP-1 receptor agonists, and TZDs in improving TT, FT, and SHBG, which was in accordance with our previous study (44). However, the meta-analysis done by Niafar et al found that TT decreased significantly after 3 months of GLP-1 receptor agonists treatment (42), and Li et al found that TZDs were superior to metformin in reducing FT after 12 weeks of treatment (45). Our results were not consistent with those of Niafar et al and Li et al, which may be due to the fact that the inclusion criteria limited weight and age, so our finding was more suitable for overweight adult PCOS. No previous meta-analysis had compared the difference between the combination of 2 insulin sensitizers and monotherapy in improving hyperandrogenemia. Our TMA revealed that both metformin combined with TZDs and metformin combined with GLP-1 receptor agonists were superior to metformin alone in improving TT and SHBG, while metformin combined with GLP-1 receptor agonists was superior to GLP-1 receptor agonists alone in improving TT, FT, and SHBG. The NMA showed similar results in improving FT and SHBG. The SUCRA curve revealed that metformin combined with GLP-1 receptor agonists was the best intervention for improving FT and SHBG. However, for decreasing TT, NMA showed no significant difference between the 5 interventions. The difference between TMA and NMA was likely due to the insufficient statistical power, because only 2 to 4 RCTs were included in each direct pairwise analysis. Because of the limitation of measurement technology, it is often difficult to accurately determine the concentration of FT, so free testosterone index (FAI) is often preferred to evaluate the level of active androgen production in vivo (46). Unfortunately, only 3 studies reported the changes of FAI, so it was impossible to carry out meta-analysis since the number was too small. Elkind-Hirsch et al (15) found that compared with metformin, metformin combined with exenatide significantly reduced FAI, while Jensterle et al (18, 19) found no significant FAI difference between metformin and liraglutide. Although the role of AND in evaluation of PCOS was unclear, it is sometimes elevated in PCOS (47). Both TMA and NMA revealed that metformin combined with GLP-1 receptor agonists was more effective than metformin or GLP-1 receptor agonists alone.

For glucose metabolism, metformin can inhibit the gluconeogenesis of hepatic glycogen, increase glucose uptake and utilization by the peripheral tissues, and improve hepatic insulin sensitivity in patients with PCOS (47). Guo found that GLP-1 receptor agonists can directly increase insulin sensitivity in fat, muscle, and liver tissues (48). Moreover, metformin can potentially enhance the effect of GLP-1 receptor agonists (36). Treatment with TZDs has been shown to improve peripheral insulin sensitivity and enhance insulin effects on skeletal muscle and adipose tissue without any direct effect on pancreatic insulin secretion (37). Both TMA and NMA revealed that metformin combined with GLP-1 receptor agonists was superior to GLP-1 receptor agonists in improving FG. Moreover, the SUCRA curve illustrated that metformin combined with GLP-1 receptor agonists was the best intervention for improving FG. Our NMA showed no significant difference between any of the 5 interventions in decreasing fasting insulin.

Obesity is a basic feature of 60% to 70% women with PCOS, and a loss of 5% to 10% of body weight has been shown to improve reproductive and metabolic outcomes in these women (49). Metformin may normalize appetite in obese women with PCOS and it may also enhance the expression of GLP-1 receptors (50, 51). GLP-1 receptor agonists bind with the receptor in the arcuate nuclei of the hypothalamus to inhibit appetite, increase the sensation of satiety, and reduce food intake, in addition to delaying gastric emptying and bowel movements and reducing body weight (52, 53). TMA showed that the combination of metformin and GLP-1 receptor agonists was better than metformin alone and metformin combined with GLP-1 receptor agonists was ranked best among the 5 regimens for WC reduction. Moreover, both TMA and NMA revealed that TZDs were inferior to metformin, which was consistent with the previous study (45), and the negative effect of TZDs on weight may be related to the fluid retention and increased appetite (54).

Unfortunately, only a few of the 14 studies included in our analysis studied improvements in ovulation and hirsutism, so it is impossible to form a NMA. Wang et al (27) and Li (22) revealed that, compared with metformin, metformin combined with TZDs could significantly increase ovulation rate. Elkind-Hirsch et al (15) found that metformin combined with GLP-1 receptor agonists was superior to GLP-1 receptor agonists and metformin alone in increasing ovulation rate. In terms of improving hirsutism and acne, Wang et al (27), Jensterle et al (18), and Ortega-Gonzalez et al (24) revealed that metformin, GLP-1 receptor agonists, TZDs, and metformin combined with TZDs could significantly improve hirsutism, but there were no statistical differences among the interventions. Ortega-Gonzalez et al (24) found that both metformin and TZDs could significantly improve acne, and Liang et al (23) showed that acne scores decreased significantly after 12 weeks of treatment with (in descending order) metformin, TZDs, and metformin combined with TZDs.

In terms of adverse events, the most common adverse effects of the pharmacologic therapies were headache and abdominal pain with metformin, GLP-1 receptor agonists, and TZDs; hypoglycemic events and gastrointestinal side effects (such as nausea, heartburn, vomiting, and diarrhea) were common with the combination of metformin and GLP-1 receptor agonists. Both GLP-1 receptor agonists and TZDs may cause insomnia, and GLP-1 receptor agonists may cause rash at the injection site and constipation; drug-specific adverse reactions associated with TZDs include mastopathy, muscle cramping, and peripheral edema. Most of the adverse effects were mild and resolved after a few weeks of treatment. Only rash at the injection site of GLP-1 receptor agonists was serious, causing 1 participant to withdraw from the trial (18).

This is the first NMA comparing efficacy and safety of both monotherapy and combinations of different insulin sensitizers in the management of overweight women with PCOS. The application of the NMA adds the ability to address the comparisons that are not powered in the TMA. All the original studies used a randomized controlled study design, which greatly reduced the likelihood of recall and selection bias. Since no prior meta-analysis has focused on the improvement of menstrual frequency, hyperandrogenemia, glucose metabolism, and obesity-related parameters simultaneously, our study is more complete.

The present study has several limitations. First, very few clinical studies have been conducted on overweight women with PCOS, and still fewer studies met the inclusion criteria of the current analysis. Most studies included had small sample sizes, and “grey literature” was not included, causing high heterogeneity and possible selection bias. Second, publication bias may exist, since studies with significant results are more likely to be published. However, we have attempted to address this issue by retrieving all the available studies, and we created funnel plots to assess any bias that may have arisen from this source. Third, the study populations differed, including European, Asian, and American populations. Fourth, the studies using GLP-1 receptor agonists compared with metformin could not adopt a blinding method. Fifth, the dosing regimens and follow-up durations were not consistent among the different studies, which may have affected the clinical efficacy. Finally, the methodology was limited in some studies, and the units of the evaluation indices were not the same. However, the conclusions and limitations of this study may provide some directions for the design of new trials.

## Conclusion

For overweight women with PCOS, both metformin combined with GLP-1 receptor agonists and metformin combined with TZDs appear superior to monotherapy in improving hyperandrogenemia. Metformin combined with TZDs could be particularly effective in promoting the recovery of menstruation. Metformin combined with GLP-1 receptor agonists has additional advantage in improving fasting glucose when compared with GLP-1 receptor agonists alone. TZDs is inferior to metformin in decreasing BMI. Overall, the available evidence is not of high quality, therefore, large-scale clinical trials are urgently needed to study different interventions with insulin-sensitive drugs in order to further guide the clinical treatment of women with PCOS.

## Summary

The aim of this network meta-analysis is to evaluate the efficacy of GLP-1 receptor agonists, TZDs or their combination with metformin on the improvement of symptoms, sex hormone and metabolic parameters in overweight women with PCOS by comparing with metformin. Our results reveal that both metformin combined with GLP-1 receptor agonists and metformin combined with TZDs appear superior to monotherapy in improving hyperandrogenemia. Metformin combined with TZDs could be particularly effective in promoting the recovery of menstruation. Metformin combined with GLP-1 receptor agonists has additional advantage in improving fasting glucose when compared with GLP-1 receptor agonists alone. TZD is inferior to metformin in decreasing BMI.

## Abbreviations

AND: androstenedione
BMI: body mass index
CI: confidence interval
CrI: credibility interval
FAI: free testosterone index
FG: fasting blood glucose
FINS: fasting insulin
FSH: follicle-stimulating hormone
FT: free testosterone
GLP-1: glucagon-like peptide-1
LH: luteinizing hormone
MD: mean difference
NA: not applicable
NMA: network meta-analysis
OR: odds ratio
PCOS: polycystic ovary syndrome
RCT: randomized controlled trial
SD: standard deviation
SHBG: sex hormone–binding globulin
SUCRA: surface under the cumulative ranking
TMA: traditional meta-analysis
TT: total testosterone
TZD: thiazolidinedione
WC: waist circumference
WMD: weighted mean difference

## Additional Information

***Disclosure Summary:*** The authors have nothing to disclose.

***Data Availability:*** All data generated or analyzed during this study are included in this published article or in the data repositories listed in References.
